# New Omics—Derived Perspectives on Retinal Dystrophies: Could Ion Channels-Encoding or Related Genes Act as Modifier of Pathological Phenotype?

**DOI:** 10.3390/ijms22010070

**Published:** 2020-12-23

**Authors:** Luigi Donato, Concetta Scimone, Simona Alibrandi, Ebtesam Mohamed Abdalla, Karim Mahmoud Nabil, Rosalia D’Angelo, Antonina Sidoti

**Affiliations:** 1 Department of Biomedical and Dental Sciences and Morphofunctional Imaging, Division of Medical Biotechnologies and Preventive Medicine, University of Messina, 98125 Messina, Italy; ldonato@unime.it (L.D.); salibrandi@unime.it (S.A.); rdangelo@unime.it (R.D.); asidoti@unime.it (A.S.); 2 Department of Biomolecular Strategies, Genetics and Avant-Garde Therapies, I.E.ME.S.T., 90139 Palermo, Italy; 3 Department of Chemical, Biological, Pharmaceutical and Environmental Sciences, University of Messina, 98125 Messina, Italy; 4 Department of Human Genetics, Medical Research Institute, University of Alexandria, Alexandria 21500, Egypt; drebtesamabdalla@yahoo.com; 5 Department of Ophthalmology, Faculty of Medicine, University of Alexandria, Alexandria 21500, Egypt; karim_nabil_ophth@yahoo.com

**Keywords:** WES, ion channels, retinal degenerations, synapses

## Abstract

Ion channels are membrane-spanning integral proteins expressed in multiple organs, including the eye. Here, ion channels play a role in several physiological processes, like signal transmission and visual processing. A wide range of mutations have been reported in the corresponding genes and their interacting subunit coding genes, which contribute significantly to a wide spectrum of ocular diseases collectively called channelopathies, a subgroup of inherited retinal dystrophies. Such mutations result in either a loss or gain-of channel functions affecting the structure, assembly, trafficking and localization of channel proteins. We investigated the probands of seven Italian and Egyptian families affected by not completely defined forms of inherited retinal dystrophies, by whole exome sequencing (WES) experiments, and found interesting variants in already known causative genes probably able to impair retinal functionalities. However, because such variants did not completely explain the phenotype manifested by each patient, we proceed to further investigate possible related genes carrying mutations that might complement previously found data, based on the common aspect linked to neurotransmission impairments. We found 10 mutated genes whose variants might alter important ligand binding sites differently distributed through all considered patients. Such genes encode for ion channels, or their regulatory proteins, and strictly interact with known causative genes, also sharing with them synaptic-related pathways. Taking into account several limitations that will be resolved by further experiments, we believe that our exploratory investigation will help scientists to provide a new promising paradigm for precise diagnosis of retinal dystrophies to facilitate the development of rational treatments.

## 1. Introduction

Inherited retinal dystrophies (IRDs) consist of a heterogeneous group of genetic disorders which share a progressive degeneration of the retina, followed by a significant impairment or loss of vision [1]. Such retinopathies represent one of the main causes of low vision and blindness at young ages, affecting more than two million people worldwide [2]. Nowadays, both syndromic and nonsyndromic forms of IRDs are known, with the second representing the most common and the most complex. The best-known syndromic forms are Usher syndrome, Leber congenital amaurosis (LCA) Bardet-Biedl syndrome and NARP (neuropathy, ataxia, and retinitis pigmentosa) syndrome [3], while retinitis pigmentosa (RP) represent the best non-syndromic group [4,5,6,7]. IRD age of onset and progression rate are influenced by numerous factors whose inheritance patterns are the most important. To date about 150 genes are known to be causative of IRDs (Retina Information Network; http://www.sph.uth.tmc.edu/RetNet/), and new ones are discovered day by day, as well as their regulating elements [8,9,10,11,12,13]. Variants in these genes are inherited as autosomal recessive (the most common kind of inheritance), autosomal dominant and X-linked patterns [14]. Mutations in a specific gene can give rise to distinct IRD phenotypes of variable severity, progression and mode of inheritance. As a consequence, establishing a reliable genotype-phenotype relationship is rarely possible [15]. Causative genes are involved in most of molecular mechanisms related to retinal function, among which the most recently associated is the synaptic activity of retinal cells mediated by different ion channels. 

### The Physiological Role of Main Ocular Ion Channel Types and Their Association to Eye-Related Diseases

Relevant studies have focused on retinal ion channel localization and distribution to figure out their role in vision pathophysiology and evaluate the effects that possible mutations could exert on the phenotype [16]. Most voltage-gated channels are localized in photoreceptors and a large number of them are distributed in horizontal, bipolar, interplexiform, amacrine and retinal ganglion cells. 

Both ligand and voltage dependent Ca^2+^, K^+^, and Cl^−^ channels in the retinal pigmented epithelial (RPE) cells contribute to retinal health, modulating volume regulation, secretory activity and transepithelial ion transport [17]. 

K^+^ channel activity controls the genesis action potential in excitable cells, as well as secretory activity and transport in nonexcitable cells. All subtypes of K^+^ channels (voltage-gated K^+^ channels (K_v_), inwardly rectifying K^+^ (K_ir_) channels, Ca^2+^-activated K^+^ channels and leak K^+^-channels) are essential in the retinal cytotypes, especially in the retinal ganglion cells (RGCs), to modulate the resting membrane potential and cell excitability [18], and in the RPE cells due to their interaction with the photoreceptor and ionic composition of the subretinal space [17]. 

The passive flow of Cl^−^ in retinal tissues is really important in RPE, where it is mediated by specific channels (the cystic fibrosis transmembrane conductance regulator (CFTR), high-conductance, voltage-gated, volume-regulated, Ca^2+^-dependent channels) that modulate the ionic composition [19]. However, the most common type of retinal ion channel is the cyclic nucleotide-gated (CNG) cationic channel, which mediates phototransduction in both photoreceptors by regulating ligand-dependent homeostasis (Ca^2+^ and Na^+^) [20].

Mutations in all these channels can lead to loss, gain or alteration of function [21]. Indeed, it has been seen that over 100 different nonsense, missense or frameshift mutations in the gene encoding the calcium channel voltage-dependent L type alpha 1F subunit (Cav1.4) can lead to decreased synaptic output from rods, also determining congenital stationary night blindness [22]. This gene is exclusively expressed in the photoreceptor synaptic terminals [23]. In addition, the calcium channel voltage-dependent L type alpha 1D subunit (Cav1.3) is localized in the photoreceptor inner segments and in the synaptic terminals [24]. Even if the role of Cav1.3 in vision is still unclear, impairments to Cav1.3 are known to be associated with Usher syndrome [25]. Furthermore, the role that chemical mediators play on ion channel modulation should not be underestimated. Mutations in the GUCY2D gene, encoding guanylyl cyclase 1, are known to be involved in cone-rod dystrophy-6 and Leber congenital amaurosis [26]. 

In this work we performed whole exome sequencing (WES) analysis of seven nonrelated patients affected by unknown forms of IRDs, showing similar and well-known phenotypes, but highlighting different subfeatures. Such peculiarities could not be sufficiently explained by identified causative mutations, and variants found in genes involved in retinal synapse modulation probably represent the real discerning factor.

## 2. Results

### 2.1. Clinical Examination of Family Probands Highlighted the Possible Impairment of Retinal Neurotransmission 

Ophthalmological features of the family probands were typical of those associated with IRDs, such as night blindness from birth, and progressive loss of visual field and visual acuity.

Fundus examination showed tapetal retinal degeneration, macular pigmentary dystrophy, pale disc and retinal pigmented epithelium atrophy. The most intriguing aspects regarded the altered phenotype related to electric stimuli, which slightly differentiate each proband. ERGs and VEPs revealed a generalized cone dysfunction with rod involvement (photopic and scotopic extinct ERGs), with a delay and a decrease in visual response (VEPs with reduced amplitude and increased latency). Furthermore, most probands described the vision of flash lights and white flies, that probably correspond to retinal neurotransmission alteration, suggesting the possible involvement of ion channels. Such a hypothesis is corroborated by diffuse hearing impairments and by effectiveness of particular treatments to which several probands are subjected, such as electrostimulation and magnetotherapy, or diet characterized by specific foods rich in important ions such as K^+^. Detailed clinical features of analyzed family probands are available in Table 1.

### 2.2. Whole Exome Sequencing Data Analysis Revealed More than One Known Causative Variant of Retinal Dystrophies in Each Proband

A sequencing run of all samples outputted a mean of 120 million reads. About 92% of total reads showed a Phred score > 30. Of these, 80% were on-target reads. Around 48,000 variants were detected for each sample. Statistics of the sequencing run are available upon request, while detailed variant calling results are presented in Table S1. Found variants were then filtered as described in the methods section and, with the help of HGMD Pro database, it was possible to find causative variants in genes known to be causative of different retinal degenerations such as cone-rod dystrophy, Usher syndrome, congenital stationary night blindness, Bardet-Biedl syndrome, choroideremia and retinitis pigmentosa. Each proband carried several causative variants of different retinal dystrophies (Table 2).

### 2.3. The Complex Genotype-Phenotype Association Suggested the Possible Involvement of Modifier Genes Encoding Ion Channels

Even if found causative variants matched with inheritance patterns, several aspects of the probands’ phenotype remained unclear. In particular, the positive effects exerted by electrostimulation, magnetotherapy and diet K^+^ supplementation, as well as the vision of white flies, could not be explained by molecular and physiological mechanisms mediated by proteins encoded by detected mutated genes. 

Thus, we realized the enrichment of mutated genes for each exome and, after clustering, we found that the cluster presenting the highest number of altered genes was related to ion channels structure and activity. In detail 10 genes, with variants differently distributed through all probands, are known to be expressed in the retina (data from GeneCards database), contributing to the neurophysiology of this nervous portion of the eye (Table 3). All considered variants carried by these genes were confirmed by Sanger sequencing (data available upon request).

### 2.4. Biochemical Analyses Highlighted Possible Altered Chemical-Physical Features in Mutated Channels

The biochemical approach realized by NovaFold and Protean 3D software suggested a huge decrease of molecular weight in mutated CLIC5 (ΔMW = 34.094 g/mol), CHRNA7 (ΔMW = 32.637 g/mol) and CACNA1A (ΔMW = 7.196 g/mol). Moreover, the presence of detected variants determined an inversion of net charge (pH = 7) in CHRNA7 (from -6.91 to +0.21), a significant increase in CLIC5 (from −29.41 to −22.62) and a relevant decrease in PIEZO1 (from −1.22 to −4.13). However, the most interesting feature highlighted by the initial phase of structural analysis was the instability index, which predicted the regional instability by estimating the weighted sum of dipeptides that occur more frequently in unstable proteins when compared to stable proteins. Both CLIC5 and CHRNA7 showed a significant increase of instability index (the first from 45.83 to 56.84, the second from 44.56 to 47.42), denoting a serious alteration of their folding and, probably, of their activity. All the other mutated proteins, instead, manifested little decrease of their instability value, a scenario that could suggest a reduction of folding flexibility, which might also alter their function.

Details of previously described parameters, as well as other qualitative ones such as hydropathy and aliphatic features, are available in Table 4.

### 2.5. 3D Structure Analysis of Mutated Channels Showed the Deletion or the Addition of Ion-Binding Sites

Although only a few of mutated encoding-channel genes clearly evidenced biochemical differences that suggested the possibility of a compromised function, the 3D structural analysis showed that the real alteration could lie in binding site differences. 

**Figure 1:**
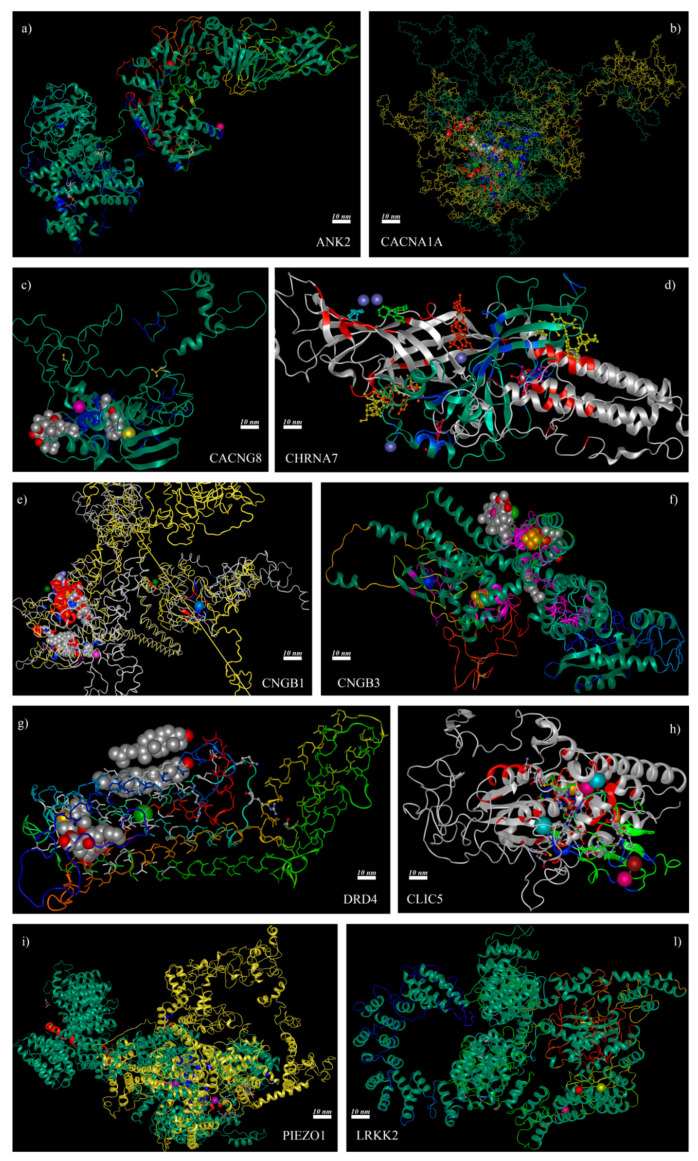
3D structural analysis of mutated channels or related proteins encoded by candidate genes. The mixed analysis of NovaFold and Protean 3D permitted evaluation of the alteration of binding sites in mutated ion channels or related proteins. Each box represents the alignment of wild-type and mutated forms of each considered predicted protein structure. Blue ribbons = mutated binding sites, red ribbons = wild-type binding sites. (**a**) Red sphere = Ca^2+^, light purple sphere = Mg^2+^. (**b**) Green sphere = Ca^2+^, dark purple sphere = Mg^2+^, (**c**) Yellow sphere = Ca^2+^, light purple sphere = Zn^2+^. (**d**) Dark purple = NH_2_. (**e**) Green sphere = Mg^2+^, blue sphere = K^+^, light purple sphere = Ca^2+^. (**f**) Green sphere = Ca^2+^. (**g**) Green sphere = Na^+^. (**h**) Turquoise sphere = Ca^2+^, yellow sphere = Fe^2+^, light purple sphere = Mn^2+^, red sphere = Zn^2+^. (**i**) Light purple sphere = Ca^2+^, pearl grey = Mg^2+^. (**l**) Yellow sphere = Ca^2+^, light purple sphere = Mg^2+^, Red sphere = selenium.

The most serious consequences emerged from truncated proteins encoded by *CHRNA7* and *CLIC5*, which lost almost all binding sites of wild-type form except one (for NH_2_ in CHRNA7 and for Ca^2+^ in CLIC5, respectively) that shifted from its original position. Three proteins showed the loss or introduction of new binding sites in their mutated form, along with positional shifting of all others: CACNA1A lost its Mg^2+^ binding site and shifted Ca^2+^ one, CNGB1 showed the addition of Ca^2+^ and K^+^ binding sites as well as the shifting of the Mg^2+^ one, PIEZO1 highlighted the introduction of a Mg^2+^ binding site and the shifting of the Ca^2+^ one. All the other ion channel-related or encoding genes showed no alterations in binding sites between wild-type and mutated forms, but identified variants resulted close to or near protein regions presenting binding sites, with the possible impairment of protein function. Details of binding site differences between wild-type and mutated forms of considered proteins are available in Table 5 and Figure 1, which also show evidenced ligands and 3D models of aligned proteins.

### 2.6. Pathways Analysis of Ion Channel-Related Mutated Genes Suggested a Complex Regulation of Synaptic Transmission Associated to Light Stimuli

All clustered pathways obtained from enrichment of ion channel-related genes by Cytoscape and its plugins ClueGO and CluePedia resulted in statistical significance (Bonferroni step-down corrected *p*-values < 0.001). 

**Figure 2:**
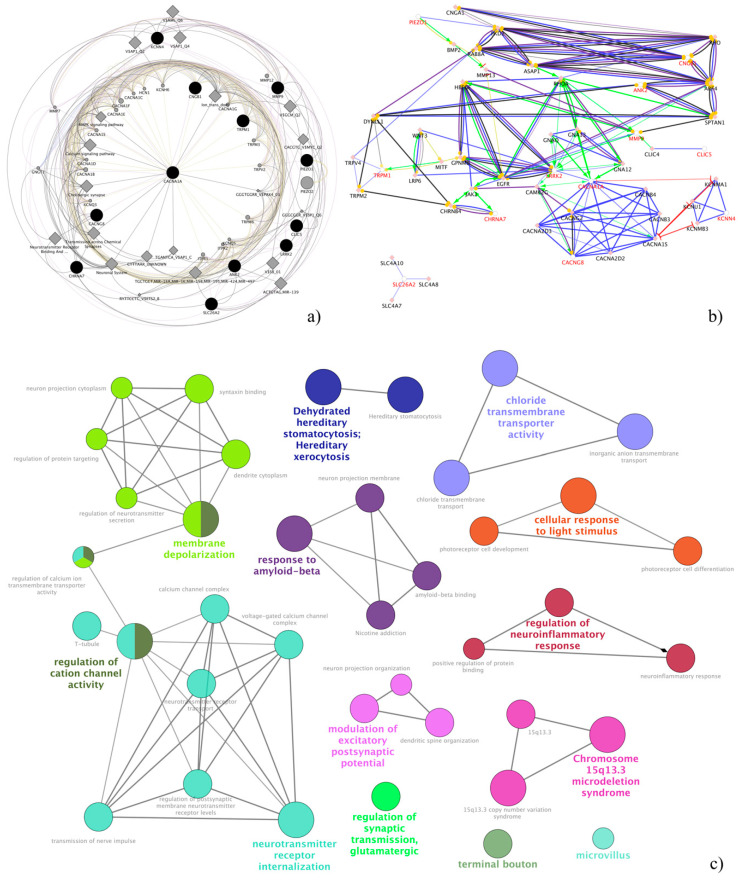
Pathway analysis by Cytoscape and its plugins. The spherical graph (**a**) represents the connections between analyzed ion channel-related genes computed by GeneMANIA, while (**b**) and (**c**) show pathways/sub-pathways and interactions computed by ClueGO and CluePedia plug-ins, respectively. It is clear that regulation of neurotransmission represented the most relevant macropathway.

Among them, seven pathways showed that the most altered functions involving these genes could deal with a complex regulation of neurotransmission related to visual physiology. In details, they were membrane depolarization, regulation of cation channel activity, neurotransmitter receptor internalization, regulation of glutamatergic synaptic transmission, modulation of excitatory post-synaptic potential, regulation of neuroinflammatory response, cellular response to light stimuli and chloride transmembrane transporter activity (Figure 2). Further features on significant pathways, especially on subpathways, are available in Table S2.

### 2.7. Ion Channel-Related Mutated Genes and Known Causative Genes of Retinal Dystrophies Suggested a Complex Regulation Network of Interactors

**Figure 3:**
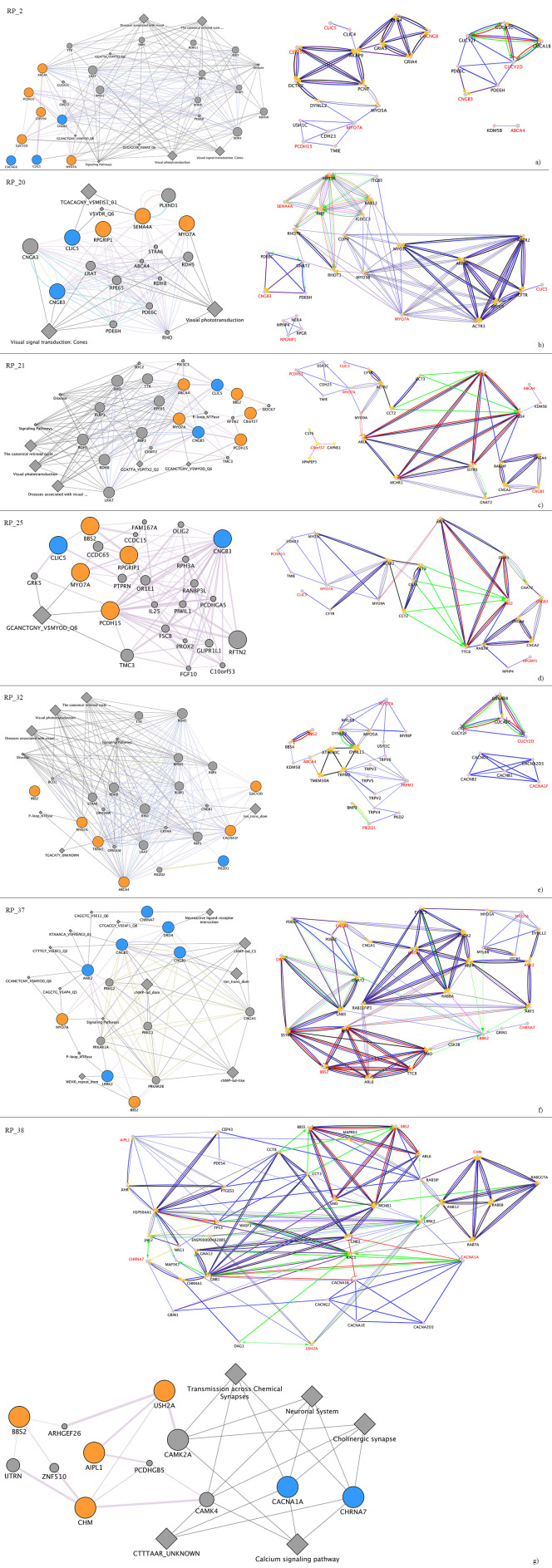
Interactions between retinal dystrophies, known causative genes and candidate ion channel-related genes. GeneMANIA (left part of each box) and CluePedia (right part of each box) network produced after analyses on both groups of genes for each patient (**a**–**g**) evidenced strong interactions between all considered elements, suggesting a possible mutual regulative activity. GeneMANIA symbol legend: orange node = known causative gene; light blue node = candidate ion channel-related gene; grey node = bridge gene; grey diamond = shared pathway or transcriptional factor; indigo edge = colocalization; dark pink edge = physical interactions; golden yellow edge = shared protein domains; antique pink = coexpression; orange edge = predicted interaction; grey edge = consolidated pathways; light blue = shared pathways. CluePedia symbol legend: red text = both causative and ion channel-related candidate genes; green edge = activation; blue edge = binding; dark purple edge = catalysis; golden yellow edge = expression; red edge = inhibition; light purple edge = ptmod; black edge = reaction.

Finally, GeneMANIA and CluePedia network analyses of known causative genes mutated in each considered patient and ion channel-related candidate genes evidenced a general co-expression and colocalization of all these genes, in certain cases also corroborated by genetic and physical interactions. Very interestingly, most of them were also involved in common regulation pathways, even if frequently of unknown type (Figure 3).

## 3. Discussion

Today is well known how genetic mutations contribute significantly to the wide spectrum of ocular channelopathies [27]. With the advances in sequencing and bioinformatics technologies, screening mutations across these genes, as well as their effect on particular channel protein functions, have become possible [28]. Research on the biophysical and biochemical properties of ion channels has revealed the fundamental processes responsible for the selective nature of these proteins to understand normal physiology [29]. However, the most challenging aspects of ion channels are represented by their not totally clear role in modulation of clinical phenotypes [30]. All ion channels are multimeric in nature and function in networks with several other regulatory proteins, but it is still unknown how the ion channels themselves might act as regulatory elements [31]. One of the most intriguing cases is represented by retinal dystrophies, a very wide and heterogeneous group of eye-related pathologies characterized by a multigenic etiology [32]. Different mutations have been observed so far across ocular ion channel genes, causing directly a wide range of channelopathies, each with its own genetic signature, thus making the screening of disease-causing genes crucial. Most of the mutations observed across these genes are missense, followed by frameshifts, nonsense, and splice-site mutations. Missense mutations in ion channel genes result in a single amino acid change, which is sufficient to alter the assembly/trafficking/function of encoded proteins. Nonsense mutations are the most deleterious as they result in either no, or truncated, protein production because of a premature termination codon (PTC). Several ocular channelopathies have been reported to be associated with nonsense mutations including cone dystrophy [33], congenital stationary night blindness [34], achromatopsia [35], bestrophinopathies [36] and retinitis pigmentosa [37]. In order to highlight the complementary role of ion channel-encoding genes mutations in the context of retinal dystrophies caused by other main causative genes, we analyzed a cohort of patients affected by different forms of retinal degenerations, characterized by particular phenotypes not completely due to principal genes. Such phenotypes, interestingly, seemed to be linked to neurotransmission alterations, probably related to ion channel impairments.

Patient RP_37 showed causative homozygous variants in genes related to Usher Syndrome (*MYO7A*) [38] and Bardet-Biedl Syndrome (*BBS2*) [39]. However, damaging effects of variants in both genes were not sufficient to explain serious alterations in color discrimination together with the extinction of scotopic signals, already in childhood. Mutations in *LRRK2* were associated with better color discrimination [40] but, probably due to the contemporary presence of variants in *DRD4* and *CHRNA7*, as well as the homozygous one in *CNGB3*, cone neurotransmission was prevalent. As can be seen in Figure 3a, most of the cited mutated genes are able to activate *LRRK2* in physiological conditions. Thus, an impairment of dopaminergic transmission mediated by LRKK2, CHRNA7 and DRD4, especially at the retinal ganglion cell level, might increase cone functional impairment already determined by the mutated CNGB3 and by the causative BBS2. This scenario could be corroborated by previous rod damage caused by MYO7A, and worsened by mutations carried by ANK2 and CNGB1, abundantly expressed in rods. Interestingly, the significant deafness phenotype might be intensified by the variant in ANK2, also expressed in the cochlea and fundamental for the mechanosensitive response for hearing [41].

An interesting case is also represented by patient RP_38, who presented disease mutations in a gene causative of Usher Syndrome (*USH2A*), Bardet-Biedl Syndrome (*BBS2*), retinitisp (*AIPL1*) [42] and choroideremia (*CHM*) [43]. If mutated, USH2A and BBS2 could explain the deafness. However, the mutations affecting the two other genes are insufficient to determine retinitis pigmentosa and choroideremia. The previously cited genes could not explain the hereditary pattern of retinal dystrophy affecting the patient (choroideremia is an X-linked disease and AIPL1 is related to recessive forms of retinal degenerations), and cerebellar atrophy is of unknown origins. The latter could be linked to a variant carried by the *CACNA1A* gene encoding for the P/Q (CaV2.1) calcium channel and known to be associated with spinocerebellar ataxia, which implies eye-related impairments with late onset [44,45,46,47]. Thus, the young age of the patient means that such earliness might be determined by the involvement of other genes. As represented in Figure 3b, it is possible that the central node of biochemical pathways made of LRRK2 could be found in an impasse, due to mutation of surrounding genes *USH2A*, *CHM* and *CACNA1A*. Furthermore, the reduced visual acuity, already caused by mutations in AIPL1 and BBS2, might be worsened by alterations of the cholinergic pathway at the retinal ganglion cell level mediated by CHRNA7 [48,49,50,51].

The case of RP_2 highlighted the most intriguing synaptic hypothesis. All causative variants were identified in the condition but generally at the basis of autosomic recessive forms of retinal degenerations (Usher syndrome, RP, cone-rod dystrophy). Even if the phenotype could be explained by an allelic heterogeneity typical of many forms of retinal dystrophies, the phenomenon of white flies remains unclear. Excluding a vitreous detachment problem, on the basis of specific instrumental examination and on the basis of intrinsically characteristics of vitreous myodesopsias (typically black), this sign might be derived from a synaptic alteration in the inner layer of the retina. Here the potentially misfolded TARP-γ8 encoded by mutated *CACNG8* might reduce the surface expression of AMPAR on retinal ganglion cells [52,53]. Such impairment, together with an increased Ca^2+^ permeability consequent of the mutations carried by CLIC5 and CNGB3, might lead to an initial transient neuronal hyperexcitability (manifested by the white flies). This event might be followed by the death of the retinal ganglion cells themselves, due to excitotoxicity, as already revealed by other neurodegenerative pathologies [54].

As the in previous case, HGMD Pro found heterozygous variants for patient RP_20 in genes associated with particular forms of retinal degeneration inherited by an autosomal recessive pattern. An ophthalmological examination highlighted that the main damage could be carried by rods. The abnormalities in depth and spatial perception might be linked to the compromised nervous function from retinal ganglion cells, as well as from the sensory cortex, cerebellum and vestibular system. Even if the latter might be determined by the mutated form of MYO7A, the other neurotransmission impairment involving superior centers might reflect the same scenario seen for RP_2, with a neuronal excitotoxicity induced by mutations carried by *CLIC5* and *CNGB3*.

Patient RP_21 had several variants that are sufficient to explain cone impairment. These variants comprise two heterozygous causative variants of cone-rod dystrophy, two heterozygous Usher Syndrome causative variants and one homozygous variant linked to the Bardet-Biedl phenotype. Challenging signs, which were not satisfactorily clear, consisted of the reduced amplitude of both pattern and flash VEPs, as well as the positive effects induced by magnetotherapy and electrostimulation. This picture suggested that the clinical phenotype showed by the patient might be related to complications at the post-retinal level, similar to optic nerve ischemic neuropathy [55]. Such alterations could induce axonal degeneration and loss of retinal ganglion cells by apoptosis. This scenario might be caused by the already cited mutated forms of CLIC5 and CNGB3, which might induce Ca^2+^-mediated excitotoxicity and reactive oxygen species (ROS) production [56]. Therefore, probably due to functional alterations carried by ion channels, electromagnetic therapies might act as neuronal pacemaker, modulating ion flux in survived retinal ganglion cells.

The distinct genetic feature of RP_25 consisted of the presence of a heterozygous causative variant of cone-rod dystrophy in the *RPGRIP1* gene, encoding a scaffolding protein required for the normal intracellular vesicular trafficking [57,58]. As for the other patients, variants are carried by ion channel-encoding genes as *CLIC5* and *CNGB3*. However, in this case their induced neurotransmission alteration is probably worsened by the mutated *RPGRIP1*. When mutated, it might reduce the transport of ion channel subunits towards cellular membranes of retinal cells, determining the increased latency of both pattern and flash VEPs.

Very particular was the case of patient RP_32. His phenotype was mostly associated with several variants carried by genes causative of various dystrophic forms already present in other patients. In addition, two unique variants carried by *TRPM1* and *CACNA1F*, known to cause congenital stationary night blindness were uniquely identified in the patient. However, dizziness and the improvement of visual acuity after the intake of bananas remained unclear. This uniqueness of phenotype might be related to a mutation carried by the *PIEZO1* gene, which encodes for a mechanically-activated ion channel that links mechanical forces to biological signals [59]. Piezo channels are mechanosensitive, nonselective cation channels that mediate force detection in eukaryotic cells, transducing mechanical stimuli in several different physiological processes [60,61,62]. *PIEZO1*, normally expressed in several parts of nervous system such as inner ear and optic nerve, might alter the stimuli perception in these organs when mutated [63,64]. Furthermore, the K^+^ intake derived from a particular diet might favor the onset of receptor graded potential and the hyperpolarization phase of action potential in retinal nervous cells, further compromising neurotransmission and leading to signal aberrations, such as flashes of light or white flies. 

## 4. Materials and Methods 

### 4.1. Clinical Data and Sample Collection

**Figure 4:**
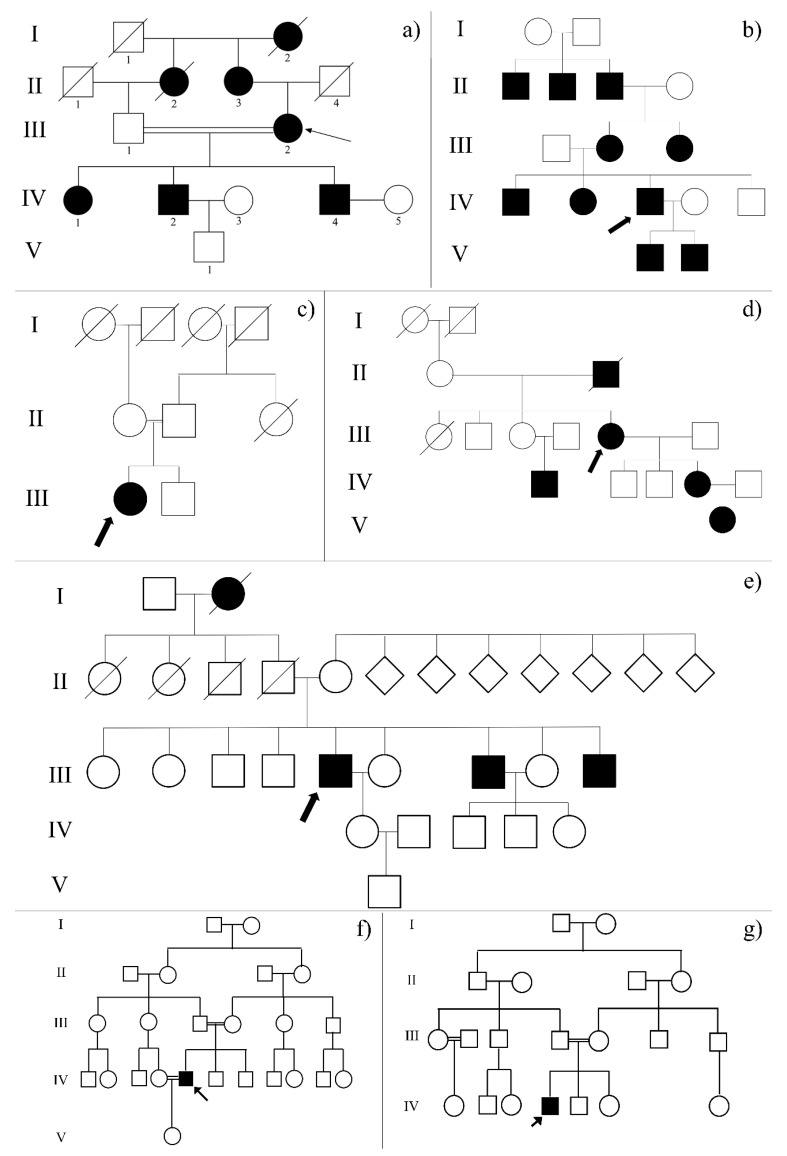
Pedigree of the analyzed families with unclear phenotypes related to specific forms of retinal dystrophies. Figure represents families of RP_2 (**a**), RP_20 (**b**), RP_21 (**c**), RP_25 (**d**), RP_32 (**e**), RP_37 (**f**) and RP_38 (**g**). The affected (black fill) and unaffected (no fill) members are shown. Arrow = proband; circle = female; square = male; double line = blood relatives; crossed = dead.

The probands of seven families, five of Italian origins (here referring to the ID assigned by our laboratories: RP_2, RP_20, RP_21, RP_25 and RP_32) and two from Egypt (RP_37 and RP_38), presented to “A.O.U. G. Martino” of Messina, Italy, and to the ophthalmological clinic at the Human Genetics Department, Medical Research Institute of Alexandria University, with clinical diagnosis of different forms of IRDs (Figure 4).

The ophthalmic examination performed on patients foresaw best-corrected visual acuity (BCVA) according to Snellen E chart. Fundoscopy (TRC- 50DX, Topcon Inc, Japan), color sensitivity was tested by Ishihara plates and macular structure observation by spectral-domain optical coherence tomography (SD-OCT, Cirrus HD-OCT, Zeiss Meditec, German). Perimetry of central 30 degrees was considered for visual field function analysis (750i Humphrey VF automated perimetry, Zeiss, German). Visual electrophysiological tests, including pattern visual evoked potentials and full-field flash electroretinography, were performed by a Retiscan/Retiport system (Roland Consult, German), and performed and complied with the standard published by the International Society for Clinical Electrophysiology of Vision (ISCEV). The diagnosis of IRD was followed by symptoms of night blindness and progressive loss of peripheral vision. Typical signs of ocular fundus, visual field and visual electrophysiological examination were also considered. 

Furthermore, genetic counseling permitted determination of the inheritance pattern of each form, giving important suggestions on possible causative genes that should be screened for variants (Figure 4).

DNA was extracted by peripheral blood and purified by the QIAamp DNA Blood Mini Kit (Qiagen). Quali-quantitative assessment was then performed by Qubit fluorometer (Thermo Fisher Scientific).

The study involved human participants and was approved by the local Ethics Committee “A.O.U. G. Martino” (Date: 23 June 2017; Number: 858). All patients agreed with their involvement in the study and written informed consent was obtained, also for minors.

### 4.2. Whole Exome Sequencing and Data Analysis

Paired-end libraries were generated by the SureSelect XT HS Reagent Kit (V6) (Agilent) kit and sequenced on a NovaSeq 6000 Illumina platform. Produced raw data were quality checked by the FastQC (v.0.11.7) tool (http://www.bioinformatics.babraham.ac.uk/projects/fastqc) and filtered according to Phred score value (reads with Phred score < 30 were trimmed, along with adaptor sequences). Selected reads were then processed by Qiagen CLC Genomics Workbench v.20.0.4 [65] and DNASTAR Lasergene Suite v.17.1. The analytic pipeline foresaw the alignment to the GRCh38 Human Reference Genome, followed by duplicate read removal, InDel realignment and base recalibration before variant calling. Finally, found variants were annotated by ANNOVAR v.2020Oct07 tool and included databases [58].

### 4.3. Variant Filtering and Gene Prioritization

Qualitative parameters of sequence run, alignment and variant calling, along with gene biotype and variant functional classes, were the first criteria evaluated for variant filtering. In detail, protein-coding genes affected by missense, nonsense and loss of function variants were selected. Then, we checked the presence of considered variants on specific reference diagnostic databases such as ClinVar [66] and Human Gene Mutation Database (HGMD) Professional [67], trying to find variants in already known IRD causative genes. If none of these presented variants, we further analyzed VCF data looking for candidate mutated genes, prioritizing them according to the enrichment analysis based on CLINVAR, Gene Ontology (Biological Process, Cellular Component, Molecular Function and Immune System Process), INTERPRO, KEGG, REACTOME (Pathways and Reactions), WIKIPATHWAYS and CORUM 3.0 as selected ontologies, considering a GO Term/Pathway Network Connectivity (Kappa Score) = 0.4. To obtain reliable results, Bonferroni-adjusted p-values < 0.01 were considered significance thresholds in statistical two-sided hypergeometric tests performed during enrichment. 

### 4.4. Variant Validation 

Once selected, variants carried by known causative genes or prioritized candidate ones were validated by Sanger sequencing. Specific primer pairs spanning the nucleotide sequence of the variants were designed for polymerase chain reaction (PCR). PCR amplifications were carried out in a 50 µL solution containing 2 µL of each primer (10 µM), 0.8 µg of genomic, 1.5 U MyTaq DNA polymerase (Bioline, London, UK). After an initial denaturation step at 95˚C for 1 min, the samples were subjected to 35 cycles of amplification consisting of 15 sec of denaturation at 95˚C, 10 sec of annealing. The annealing temperature was optimized for each primer set. Following PCR, 5 µL of amplified product was examined by electrophoresis on a 1% agarose gel. Sanger sequencing was then performed using the BigDyeTerminator© v3.1 Cycle Sequencing Kit chemistry and run on a 3130xl Genetic Analyzer (Applied Biosystems, Thermo Fisher Scientific). Primer list, PCR thermal conditions and electropherograms are available upon request.

### 4.5. Proteomic in-Silico Analyses

In order to investigate possible effects of found variants on the coding sequence and their impact on each gene encoded protein, the DNASTAR Lasergene Protein suite was exploited. The comparative secondary structure prediction of wild-type and mutated protein sequence was realized by Protean 3D software (https://www.dnastar.com/software/protein/), also evaluating hydropathy, stability, transmembrane properties, cleavage sites for proteases and chemicals, and functional domains scanning PROSITE and InterPro databases. The prediction of tertiary structures was realized by Phyre2 [68], Raptor X [69] and the NovaFold tool based on Iterative Threading ASSEmbly Refinement (i-TASSER) hierarchical approach [70]. The selection of the most reliable 3D structures was realized on the basis of the highest computed template modeling score (TM-score near 1) and cluster size (indicative of a lower-energy conformation), together with the lowest root means deviation (RMSD near 0). The prediction of ligand binding sites was considered reliable on the basis of confidence score near 1.

### 4.6. Pathway and Subpathway Analyses

In order to highlight the mutual involvement of causative genes and ion channel candidate genes in the same eye-related pathways, as well as their interconnections, deep analyses with Cytoscape [71] and its plug-ins GeneMANIA [72], ClueGo [73] and CluePedia [74] were performed. 

## 5. Conclusions

The influence of ion channels and their modulators on the etiopathogenesis of retinal dystrophies is a very interesting field. We investigated the possible role of such proteins in a cohort of seven patients belonging to unrelated families from Italian and Egyptian populations. We first identified known causative genes carrying important variants possibly related to expressed clinical phenotypes and doing WES experiments. Afterwards, because such variants did not completely explain the symptomatology manifested by each patient, we proceeded to further investigate possible related genes carrying mutations that might complement previously found data, based on the common aspect linked to neurotransmission impairments. We focused on 10 candidate genes whose encoded product were characterized by 3D in silico structural analyses. This study highlighted possible alterations of several binding sites that might impair synaptic dialogue between all retinal cytotypes and superior nervous system centers.

Nevertheless, there are several critical limitations that should be evaluated when results from the realized analyses are interpreted. The most important is related to the fact that our investigation should be considered as a pilot study. Even if we found very interesting elements linking unexplored phenotypic signs of multigenic forms of retinal dystrophies to ion channel activity, several outcomes, especially interactions between encoded proteins and found ligands, must be further validated by experimental procedures (like luciferase mediated essays, e.g., Split Synthetic Renilla Luciferase Protein-Fragment-Assisted Complementation and BRET/NanoLuc^®^ Luciferase). Additionally, the number of evaluated families should be increased in order to reduce the bias related to a small effect size, and the genotyping of all other members of the families should be performed. Finally, obtained results could become more significant if an in vivo experiment confirmed what was observed in this work, but with the wider scenario of the whole retina in a model organism. Regarding this, it could also be interesting to investigate the role of pericytes in modulation of retinal ion channels, already investigated [75,76], but yet unexplored in relationship to retinal pathologies.

Taking into account such critical issues, we believe that our exploratory investigation will help scientists to provide a new promising paradigm for precise diagnosis of retinal dystrophies to facilitate the development of rational treatments.

## Figures and Tables

**Table 1:** Clinical features of the seven probands of the reported Sicilian and Egyptian families. Reported signs, symptoms and clinical-pathological features consisted of those most frequently observed in retinal dystrophies, even if several are characteristics of phenotypically orphan forms.

FEATURE	RP_2	RP_20	RP_21	RP_25	RP_32	RP_37	RP_38
**Age**	72	61	52	75	56	29	13
**Sex**	F	M	F	F	M	M	M
**Age of Onset**	4	8	4	24	10	9	3
**Age of First Diagnosis**	16	16	16	16	16	9	b
**Photophobia**	Yes	Yes	Yes	Yes	Yes	Yes	Yes
**Night Blindness**	Yes	Yes	Yes	Yes	Yes	Yes	Yes
**Color Vision**	Altered	Present	Reduced	Reduced	Altered	Altered	Reduced
**Hearing Involvement**	Yes	No	No	No	Yes	Yes	Yes
**Flashes of Lights**	No	No	No	Yes	Yes	No	No
**White Flies**	Yes	No	No	Yes	Yes	No	No
**ERG**	Extinct	NA	Extinct (photopic)	Near extinction (photopic); extinct (scotopic)	Extinct (both photopic and scotopic)	Extinct (scotopic)	Quite reduced (both photopic and scotopic)
**VEP**	NA	NA	Both VEP pattern and VEP flash very reduced amplitude and normal latency in both eyes	VEP pattern with huge P100 latency increase on the right, disrupted response on the left; VEP flash with bilateral latency quite increase	NA	NA	Intact visual pathways in both eyes
**Other**	Peripheral vision, bilateral nystagmus	Altered perception of depth	Electrostimulation and magnetotherapy cleared visual perception and delayed degeneration; in relax situations visual perception is improved	Nystagmus	Tunnel vision; dizziness (with loss of balance); the intake of bananas improves visual acuity, especially the white vision	Generating cousins	Generating cousins; mild cerebellar atrophy; cone-rod dystrophy clinical diagnosis

**Table 2:** Identified causative variants in the probands of the analyzed families. The HGMD professional annotation of whole exome sequencing (WES) detected variants permitted the identification of known causative variants in screened family probands. The homozygous variants are highlighted in red text.

FEATURE	PHENOTYPE	RP_2	RP_20	RP_21	RP_25	RP_32	RP_37	RP_38
***GUCY2D***	**Cone-Rod Dystrophy**	rs61750173 (p.R838H)						
						rs138836357 (p.R365W)		
***ABCA4***		rs6666652 (p.S1047I)		rs6666652 (p.S1047I)		rs6666652 (p.S1047I)		
***C8orf37***				rs36096184 (p.P19S)				
***RPGRIP***			rs10151259 (p.A547S)		rs10151259 (p.A547S)			
***MYO7A***	**Usher Syndrome**	rs1052030 (p.L16Ter)		rs1052030 (p.L16Ter)	rs1052030 (p.L16Ter)	rs1052030 (p.L16Ter)	rs1052030 (p.L16Ter)	
			rs77625410 (Y1719C)					
***PCDH15***		rs4935502 (p.D435A)		rs4935502 (p.D435A)	rs4935502 (p.D435A)			
***USH2A***								rs696723 (p.G713S)
***SEMA4A***	**Retinitis Pigmentosa**		rs41265017 (p.R713Q)					
***CEP290***		rs183655276 (p.D1413H)						
***AIPL1***								rs150427474 (p.R261Q)
***TRPM1***	**Congenital Stationary Night Blindness**					rs138886378 (p.S157F)		
***CACNA1F***						rs141159097 (p.N746T)		
***BBS2***	**Bardet-Biedl Syndrome**			rs4784677 (p.S70N)	rs4784677 (p.S70N)	rs4784677 (p.S70N)	rs4784677 (p.S70N)	rs4784677 (p.S70N)
***CHM***	**Choroideremia**							rs55741408 (p.L80F)

**Table 3:** Mutated genes related to ion channels were identified in the probands of the analyzed families. The GO annotation of family probands’ WES detected several variants carried by genes related to ion channel activity screened family probands. The homozygous variants are highlighted in red text.

GENE	GO Info	RP_2	RP_20	RP_21	RP_25	RP_32	RP_37	RP_38
*ANK2*	Required for Na^+^/K^+^-ATPase, Na+/Ca^2+^ exchanger and Beta-2-Spectrin expression; abundant in rods						I825T	
*CACNA1A*	The channel activity is directed by the pore-forming alpha-1 subunit, whereas the others act as auxiliary subunits. The isoform alpha-1A gives rise to P and/or Q-type calcium currents							Q792_Q800del
*CACNG8*	Modulates Ca^2+^ L-type channel activity and AMPAR opening	G327C; G361V						
*CHRNA7*	Subunit of nicotinic ACh channel-receptor; it forms a homo-oligomeric channel with high permeability to Ca^2+^						L166fs	L166fs
*CLIC5*	Constitutes channel with low-selectivity, also for Cl^−^	S106ter	S106ter	S106ter	S106ter			
*CNGB1*	Subunit of nonselective cationic ion channels mediated by cyclic nucleotides; abundant in rods						E370_E371del	
*CNGB3*	Beta subunit of a cationic ion channel mediated by cyclic nucleotides; abundant in cones	R781C	R781C	R781C	R781C		R781C	
*DRD4*	D4-subtype of dopamine receptors; abundant in retinal ganglion cells						D288G	
*LRRK2*	Serine/threonine kinase which phosphorylates proteins involved into neuronal plasticity, autophagy and vesicular trafficking						P1262A	
*PIEZO1*	Ion channel which boosts the intracellular entry of Ca^2+^					V250A; K1878del		

**Table 4:** Biochemical and physical change prediction between wild-type and mutated ion channel-related proteins. Analyses of biochemical and physical parameters by Protean 3D showed a global alteration of such parameters throughout all investigated proteins, reaching the highest instability in mutated CHRNA7 and CLIC5.

Gene	Wt/Mutated	MW [g/moL]	Net Charge [pH = 7]	pI	Average Hydropathy	Aliphatic Index	A₂₈₀ (ox.)	A₂₈₀ (red.)	ε₂₈₀ [M^−^¹ cm^−^¹]	Instability Index	Features
***ANK2***	Wt	165,738.9	−188.1	4.32	−0.88	60.79	0.38	0.37	61,770	72.04	EFO, CA, MG, OHX, MN, MG, NDP, MYR, CA, DCA, 43 helices, 62 strands
	I825T	165,726.94	−188.01	4.32	−0.88	60.53	0.38	0.37	61,770	71.82	86 helices, 124 strands, I825T
***CACNA1A***	Wt	225,243.55	34.65	9.14	−0.59	68.01	0.99	0.99	222,890	53.1	9SL, ANP, III, MGE, Y01, CA, MG, 6OU, 61 helices, 4 strands
	Q792_Q800del	218,047.31	36.67	9.22	−0.60	68.10	0.97	0.97	211,430	52.29	9SL, III, CA, Y01, ANP, UUU, MG, MGE, 9Z9, 6OU, 74 helices
***CACNG8***	Wt	43,312.87	11.22	9.34	−0.10	77.93	1.09	1.08	46,870	57.88	ACD, BCL, XE, III, ZN, CVM, MG, NUC, CA, CA, 11 helices, 5 strands
	G327C; G361V	43,401.04	11.16	9.29	−0.08	78.61	1.09	1.08	46,870	57.21	22 helices, 10 strands, G327C, G361V
***CHRNA7***	Wt	56,449.44	−6.91	6.02	0.08	95.10	1.76	1.74	98,320	44.56	III, MLK, DSF, ZY5, EPJ, IVM, TC9, 10 helices, 12 strands
	L166fs	23,812.21	0.21	6.95	−0.31	86.44	2.71	2.7	64,400	47.42	III, CU9, MLK, PLC, V11, 9Z0, 3 helices, 10 strands
***CLIC5***	Wt	46,502.65	−29.41	4.71	−0.69	73.32	0.95	0.94	43,780	45.83	GSH, MNB, ASC, P10, GTX, GDS, CA, 12 helices, 6 strands
	S106ter	12,409.07	−22.62	3.97	−1.21	60.66	1.20	1.20	14,900	56.84	FE2, CA, MN, FAD, OXD, OXY, ZN, ACT, MN3, 1 helix, 3 strands
***CNGB1***	Wt	139,677.76	−78.93	4.75	−0.60	75.08	1.15	1.14	159,170	65.92	CMP, ANP, 6ZL, CLA, PGW, III, CA, SF4, MG, 30 helices, 12 strands
	E370_E371del	139,419.53	−76.93	4.77	−0.59	75.20	1.15	1.14	159,170	65.49	CMP, B73, ANP, PGW, IAC, K, CLA, MG, CA, CH1, 28 helices, 11 strands
***CNGB3***	Wt	92,166.52	3.64	8.06	−0.51	82.81	1.09	1.09	100,160	43.43	CMP, PGW, CA, SF4, 78M, ANP, K, III, CLA, SF4, 29 helices, 13 strands
	R781C	92,113.47	2.58	7.81	−0.50	82.81	1.09	1.09	100,160	43.26	58 helices, 26 strands, R781C
***DRD4***	Wt	43,901.46	14.89	9.24	0.30	96.01	1.02	0.99	43,430	49.26	AQD, ERC, NA, CLR, 1WV, CLR, 2CV, 2CV, SOG, CLR, 17 helices
	D288G	43,843.43	15.88	9.31	0.31	96.01	1.02	0.99	43,430	48.81	34 helices, D288G
***LRRK2***	Wt	224,673.52	−31.59	6.06	−0.11	104.81	0.73	0.72	161,120	45.81	CA, CLA, III, III, SE, UUU, MG, UUU, UUU, III, 98 helices, 4 strands
	P1262A	224,647.49	−30.59	6.06	−0.11	104.85	0.73	0.72	161,120	45.65	196 helices, 8 strands, P1262A
***PIEZO1***	Wt	224,984.30	−1.22	7.07	0.06	101.72	1.66	1.65	370,820	51.95	ANP, 9SL, MGE, NUC, Y01, MG, III, 9Z9, CA, GRG, 79 helices, 4 strands
	V250A; K1878del	226,789.15	−4.13	6.85	0.06	101.34	1.65	1.64	370,820	51.55	MGE, III, BCL, ANP, CA, P10, GRG, CLA, MG, 104 helices, 2 strands

**Table 5:** Details of binding site differences between wild-type and mutated forms of ion channel-related proteins. All predicted structure of evaluated ion-channel related proteins showed the insertion/loss of important binding sites or a folding impairment due to the presence of detected variants. The complete explanation of complex ligands is available in the dictionary of chemical components on PDBeChem Ligand Dictionary on Protein Data Bank of EMBL-EBI site (https://www.ebi.ac.uk).

GENE	VARIANT	LIGAND BINDING SITE (Wt) [AA]	LIGAND BINDING SITE (Mut) [AA]	NOTES
*ANK2*	I825T	EF0: 1498 Mg: 1499 F2A: 1500 NDP: 1501 Ca: 1502	EF0: 1498 Mg: 1499 F2A: 1500 NDP: 1501 Ca: 1502	Binding sites for all predicted ligands identical for wild and mutated proteins
*CACNA1A*	Q792_Q800del	9SL: 1997 ANP: 1998 Y01: 1999 Ca: 2000 Mg: 2001 6OU: 2002	9SL: 1937 Ca: 1938 Y01: 1939 ANP: 1940 9Z9: 1941 6OU: 1942	Ca^2+^ binding sites almost shifted, deletion of a Mg^2+^ binding site in mutated protein
*CACNG8*	G327C; G361V	ACD: 426 BCL: 427 Ca: 430 XE: 428 Zn: 429	ACD: 426 BCL: 427 Ca: 430 XE: 428 Zn: 429	Binding sites for all predicted ligands identical for wild and mutated proteins
*CHRNA7*	L166fs	NH2: 503, 506, 509 MLK: 504 DSF: 505 ZY5: 507 EPJ: 508 IVM: 510 TC9: 511	NH2: 206 Cu9: 207 9Z0: 208–209	Deletion of several ligand binding sites in mutated protein
*CLIC5*	S106ter	GSH: 411, 415 GTT: 412 MNB: 413 ASC: 414 GTX: 416 GDS: 417 Ca: 418	Fe2: 107 Mn: 108 Ca: 109 FAD: 110 OXD: 111 OXY: 112 Zn: 113 Mn3: 114	Ca^2+^ binding sites almost shifted, creation of a several new ion binding sites in mutated protein
*CNGB1*	E370_E371del	Unknown: 1252-1264 ANP: 1265-1266 6ZL: 1267 CLA: 1268 PGW: 1269 SF4: 1270 Mg: 1271	B73: 1250 ANP: 1251 PGW: 1252 IAC: 1253 K: 1254 CLA: 1255 Mg: 1256 Ca: 1257 CH1: 1258	Mg^2+^ binding site almost shifted, creation of a K^+^ and Ca^2+^ binding sites in mutated protein
*CNGB3*	R781C	PGW: 810 Ca: 811 SF4: 812, 817 78M: 813 ANP: 814 K: 815 CLA: 816	PGW: 810 Ca: 811 SF4: 812, 817 78M: 813 ANP: 814 K: 815 CLA: 816	Binding sites for all predicted ligands identical for wild and mutated proteins
*DRD4*	D288G	AQD: 420 ERC: 421 Na: 422 CLR: 423, 425, 427 1WV: 424 WHJ: 426	AQD: 420 ERC: 421 Na: 422 CLR: 423, 425, 427 1WV: 424 WHJ: 426	Binding sites for all predicted ligands identical for wild and mutated proteins
*LRRK2*	P1262A	Unknown: 1981-1984	Unknown: /	Variant near Mg^2+^ binding site, far from Se and Ca^2+^ ones
*PIEZO1*	V250A; K1878del	ANP: 1981 9SL: 1982 Y01: 1983 9Z9: 1984 Ca: 1985	Unknown: 1999-2000 BCL: 2001 ANP: 2002 Ca: 2003 CLA: 2004 Mg: 2005	Ca^2+^ binding sites almost shifted, creation of a Mg^2+^ binding site in mutated protein

## Data Availability

RAW exomic data is available upon request.

## Supplementary Materials

Supplementary materials can be found at https://www.mdpi.com/1422-0067/22/1/70/s1. Table S1. Detailed variant calling results; Table S2. Features on significant pathways, especially on subpathways from evaluated genes by Cytoscape plug-ins.

## Abbreviations

IRDs	Inherited Retinal Dystrophies
RPE	Retinal Pigment Epithelium
RGCs	Retinal Ganglion Cells
CFTR	Cystic Fibrosis Transmembrane Conductance Regulator
LCA	Leber Congenital Amaurosis
RP	Retinitis Pigmentosa
ERG	Electroretinogram
VEP	Visual Evoked Potential
HGMD	Human Gene Mutation Database
WES	Whole Exome Sequencing
GO	Gene Ontology
PTC	Premature Termination Codon
AMPAR	AMPA Receptors
BCVA	Best-Corrected Visual Acuity
SD-OCT	Spectral-Domain Optical Coherence Tomography
ISCEV	International Society for Clinical Electrophysiology of Vision
PCR	Polymerase Chain Reaction
RMSD	Root Mean Square Deviation
